# Genotyping by Sequencing of Cultivated Lentil (*Lens culinaris* Medik.) Highlights Population Structure in the Mediterranean Gene Pool Associated With Geographic Patterns and Phenotypic Variables

**DOI:** 10.3389/fgene.2019.00872

**Published:** 2019-09-18

**Authors:** Stefano Pavan, Nicoletta Bardaro, Valentina Fanelli, Angelo Raffaele Marcotrigiano, Giacomo Mangini, Francesca Taranto, Domenico Catalano, Cinzia Montemurro, Claudio De Giovanni, Concetta Lotti, Luigi Ricciardi

**Affiliations:** ^1^Department of Soil, Plant and Food Science, Section of Genetics and Plant Breeding, University of Bari Aldo Moro, Bari, Italy; ^2^Institute of Biomedical Technologies, National Research Council (CNR), Bari, Italy; ^3^CREA Research Centre for Cereal and Industrial Crops (CREA-CI), Foggia, Italy; ^4^Department of Agricultural, Food and Environmental Sciences, University of Foggia, Foggia, Italy

**Keywords:** breeding, conservation genetics, genetic structure, genotyping-by-sequencing, lentil

## Abstract

Cultivated lentil (*Lens culinaris* Medik.) is one of the oldest domesticated crops and one of the most important grain legumes worldwide. The Mediterranean Basin holds large part of lentil biodiversity; however, no genetic structure was defined within the Mediterranean gene pool. In this study, we used high-throughput genotyping by sequencing to resolve the genetic structure of the Mediterranean *ex situ* lentil collection held at the Italian National Research Council. Sequencing of a 188-plex genotyping-by-sequencing library and bioinformatics treatment of data yielded 6,693 single nucleotide polymorphisms. Analysis of nonredundant genotypes with nonparametric and parametric methods highlighted the occurrence of five highly differentiated genetic clusters. Clustering could be related to geographic patterns and phenotypic traits, indicating that post-domestication routes introducing cultivation in Mediterranean countries and selection were major forces shaping lentil population structure. The estimation of the fixation index F_ST_ at individual single nucleotide polymorphism loci allowed the identification of distinctive alleles across clusters, suggesting the possibility to set up molecular keys for the assignment of lentil germplasm to specific genetic groups. Finally, significant associations between markers and phenotypic data were identified. Overall, the results of this study are of major importance for lentil conservation genetics and breeding and provide insights on the lentil evolutionary history.

## Introduction

Lentil (*Lens culinaris* Medik., 2n = 2x = 14) is one of the crops signing the birth of agriculture in the Neolithic Near East. Starting from the Fertile Crescent, lentil cultivation spreads westward to the Mediterranean Basin, the Nile Area, and Central Europe and eastward to Asia. More recently, lentil was introduced to North America and Australia (Cubero, 1984; Matny, 2015).

Nowadays, lentil is the third most widespread cool-season grain legume in the world, after chickpea (*Cicer arietinum* L.) and pea (*Pisum sativum* L.) (FAOSTAT data, 2017). Lentil global area and production significantly increased during the last decade, reaching 6.6 million ha and 7.6 million tons, respectively, with Canada, India, and Turkey being the main lentil producers (FAOSTAT data, 2017). Similar to other legume species, lentil increases soil fertility through nitrogen fixation and has a positive impact on soil properties and conservation (Sultani et al., 2007). In addition, lentils are of utmost importance for food security, as they represent an affordable source of dietary proteins, vitamins, and other nutrients (Pal et al., 2016).

*Ex situ* germplasm collections provide broad genetic variation to cope with future agricultural challenges, including climate changes, soil degradation, and water and land shortage (Fu, 2017). Information on phenotypic and molecular diversity is crucial for the effective setup and management of *ex situ* collections and the choice of germplasm suitable for breeding purposes. In lentil, genetic divergence between parental lines used in breeding programs is an important factor influencing genetic gains (Roy et al., 2013).

Following the advent of next-generation sequencing technologies, single nucleotide polymorphism (SNP) markers have been used in lentil to describe genetic variation of germplasm collections and link-specific markers to phenotypic traits, including seed quality, disease resistance, and micronutrient concentration (Lombardi et al., 2014; Sudheesh et al., 2016; Khazaei et al., 2016; Khazaei et al., 2017; Khazaei et al., 2018). A comprehensive investigation of lentil genetic structure identified three distinct clusters, broadly reflecting three world’s agro-ecological zones: Mediterranean Basin (also including the Nile valley from Egypt to Ethiopia), subtropical Asia, and northern temperate (Khazaei et al., 2016). Mediterranean germplasm holds large part of lentil genetic diversity (Erskine et al., 1989; Toklu et al., 2009; Lombardi et al., 2014; Khazaei at al., 2016). So far, variation in the Mediterranean gene pool could not be associated with specific geographic or phenotypic patterns.

Genotyping by sequencing (GBS) (Elshire et al., 2011), based on the sequencing of reduced-representation genomic libraries obtained by restriction enzyme digestion, is one of the most effective methods for SNP discovery and genotyping. GBS protocols have been developed for species with and without a reference genome (Lu et al., 2013; Glaubitz et al., 2014) and have been successfully applied for the characterization of germplasm collections, family-based linkage mapping, and genome-wide association (GWA) studies (Taranto et al., 2016; Pavan et al., 2017a; Pavan et al., 2017b; D’Agostino et al., 2018; Pavan et al., 2018).

The Institute of Biosciences and Bioresources of the Italian National Research Council (IBBR-CNR) holds one of the most important legume collections worldwide (http://ibbr.cnr.it/mgd/). At present, the collection includes 15,724 accessions, in most cases landraces collected from 1970s to 1990s in the Mediterranean area. In this study, we used GBS to explore the genetic diversity of 185 accessions selected within the IBBR-CNR *Lens* collection. The main aims of this study were to dissect lentil population structure and to provide a link between structure and geographic, phenotypic, and molecular variables. Genotypic and phenotypic data were finally merged aiming to detect significant associations.

## Materials and Methods

### Plant Material

The IBBR-CNR *Lens* collection includes 349 accessions, in most cases referable to local landraces collected through exploratory missions carried out from 1971 to 1993 (http://ibbr.cnr.it/mgd/). A set of 184 *Lens culinaris* accessions was selected to maximize variation with respect to geographic origin. The set mostly included Mediterranean germplasm (with this term also including the Nile valley from Egypt to Ethiopia) and Asian germplasm. In addition, four individuals obtained by self-pollination of the cultivar “Laird” were selected to provide a reference for the identification of redundant accessions (**Supplementary Table S1**).

### Genotyping-by-Sequencing Analysis

Genomic DNA was extracted from young leaf samples using the DNeasy Plant Mini Kit (Qiagen). A reduced representation library was prepared as described by Elshire et al. (2011), using the *ApeK*I restriction enzyme, and sequenced (paired ends) using the Illumina HiSeq 2500 system (Illumina). The TASSEL-Universal Network Enabled Analysis Kit pipeline (Lu et al., 2013), suitable for species without a reference genome, was used for SNP calling. Biallelic SNPs were selected and filtered for minor allele frequency (> 5%), call rate (> 80%), and inbreeding coefficient (> 0.8), using TASSEL v. 5.2.20 (Bradbury et al., 2007). Finally, the quality control procedure involved the exclusion of accessions with call rate < 80%.

### Identification of Redundant Accessions

In order to identify genetically redundant accessions, a matrix of pairwise identity by state (IBS) distance was calculated using SVS v.8.8.3 (Golden Helix). The mean and standard deviation of pairwise IBS distances calculated among four biological replicates of the cultivar “Laird” were considered to set up a threshold (mean – 3 × standard deviation) above, which two genotypes were declared redundant.

### Population Structure and Diversity Among Accessions

In order to minimize correlation among loci used to study population structure, the SNP input dataset referring to nonredundant accessions was pruned for linkage disequilibrium (r^2^ < 0.5) using SVS 8.8.3 (Golden Helix).

Analysis with the STRUCTURE (v.2.3.4) parametric model (Pritchard et al., 2000) was carried out for a number of hypothetical subpopulations (the K parameter) ranging from 1 to 10, using 10 independent runs for each K, a burn-in period of 25,000 and 100,000 Markov chain Monte Carlo iterations. The most probable number of clusters was inferred by the calculation of the ΔK statistics (Evanno et al., 2005), using the software Structure Harvester (Earl and VonHoldt, 2012). Individual samples were assigned to each subpopulation when the value of the corresponding membership coefficient (q_i_) was higher than 0.6, otherwise they were classified as admixed.

Nonparametric study of population structure was carried out using the “find.clusters” function implemented in the stats R package, returning k-means clusters ranging from 1 to 40. The optimal number of clusters was inferred by the Bayesian Information Criterion statistics. Discriminant functions were calculated by the adegenet R package (Jombart et al., 2010), using the “xvalDapc” function to assess the number of principal components to be retained.

Pairwise distances among k-means clusters were calculated using the F_ST_ and Nei’s indexes (Nei, 1972; Wright, 1978). Nei’s distances were used to build a neighbor joining tree using the poppr and ape R packages (Paradis et al., 2004; Kamvar et al., 2014). Statistical support to each tree node was obtained by performing 100-bootstrap replications.

Genetic relationships among individual accessions were assessed by the construction of a dendrogram based on the allele sharing distance and the Ward’s clustering algorithm, using the AWclust R package (Gao and Starmer, 2008).

### F_ST_ Analysis

The pairwise fixation index (F_ST_) was computed at individual SNP loci, using the formula of Weir and Cockerham (1984) and comparing individuals of each cluster with those of the remaining clusters. Boxplots were obtained to summarize the distribution of F_ST_ values for each comparison.

### Phenotyping and Statistical Correlations With Genetic Clusters

Nonredundant accessions of the lentil collection were grown at the experimental farm of the University of Bari Aldo Moro (41°01′22.1′′ N 16°54′21.0′′ E) during the growing season 2017–2018, according to a randomized block design with two replicates. Each experimental unit consisted of 10 plants arranged in a row of 1 m. The average flowering time was assessed at six different time points as the number of days after sowing in which about 30% of the plants exhibited flowers. Following harvest, plant height and the height of the flowering node were recorded. Seeds from both replicates were bulked, and a random sample of 50 seeds was taken for morphometric measurements. High-resolution scanner-based images were processed using the Image-Pro Plus 7.0 software (Media Cybernetics, USA), in order to estimate seed area, perimeter, and diameter. Significance of genotypic effects on phenotypic traits was assessed by analysis of variance. The Tukey’s honestly significant difference test was performed for the *post hoc* detection of significant pairwise differences among clusters (p < 0.05). Boxplots were obtained to highlight the distribution of phenotypic traits in individual clusters.

### Genome-Wide Association Study

A GWA study was carried out to link phenotypic traits to markers identified by GBS analysis. The multi-locus mixed model implemented in SVS 8.8.3 (Golden Helix) was used for regression, using the IBS matrix as covariance matrix of random effects. The false discovery rate correction was used to declare significant associations (p < 0.05).

## Results

### Detection of Single Nucleotide Polymorphisms

Sequencing of a multiplexed GBS library prepared from 188 lentil genotypes (**Supplementary Table S1**) generated about 467 million read pairs, corresponding to an average of about 2.4 million read pairs per sample. Raw sequence FASTQ data were deposited at the National Center for Biotechnology Information Short Read Archive (http://www.ncbi.nlm.nih.gov/sra/) database under the accession number SRR8863759.

The application of the Universal Network Enabled Analysis Kit bioinformatics pipeline (Lu et al., 2013) resulted in the identification of 410,637 SNP sites. The SNP quality control procedure, based on successive filtering steps for call rate, minor allele frequency, and inbreeding coefficient, yielded 6,693 polymorphisms. The observed transitions and transversions were 4,297 and 2,396, respectively, leading to a transition/transversion ratio of 1.79. The accessions MG43, MG136, MG141, and MG146 were excluded from further analysis, as they displayed more than 20% missing data.

### Identification of Redundant Genotypes

The distribution of pairwise IBS distances showed an approximately Gaussian tail for high IBS values (**Supplementary Figure S1**). Notably, this tail overlapped with the Gaussian distribution calculated by the mean (0.98) and standard deviation (0.06) of pairwise IBS distances among four individuals of the pure line cultivar “Laird.” Therefore, an IBS threshold of 0.96 (mean − 3 × standard deviation) was set up to identify clusters of (nearly) identical genotypes. In total, 69 genotypes were assigned to 12 clusters, in most cases formed by accessions originating from the same country (**Supplementary Table S2**). For each cluster, the genotype displaying the highest call rate was retained for further analyses.

### Model-Based Inference of Population Structure and Distribution of Genetic Clusters in the Mediterranean Area

**Figure 1:**
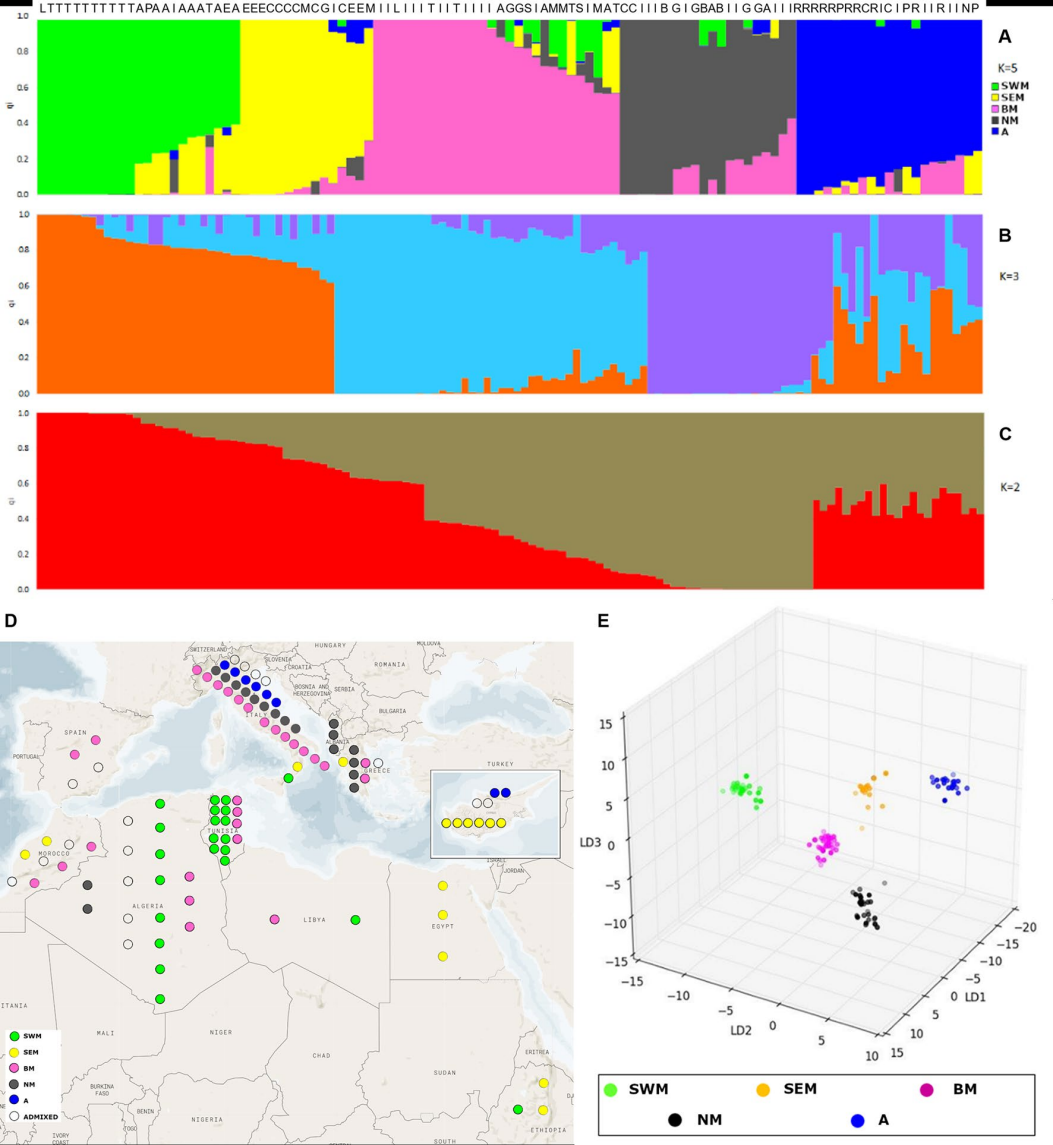
Analysis of genetic structure with the STRUCTURE admixture model and discriminant analysis of principal components (DAPC). STRUCTURE results are shown for models with two **(A)**, three **(B)**, and five **(C)** subpopulations, associated with the highest values of the Evanno’s ΔK statistics. Each individual is represented by a vertical line, which is partitioned into colored segments whose length depends on the estimated membership fraction (q_i_) in each subpopulation. Letters over the bars indicate the country of origin of individual accessions: Algeria (A), Cyprus (C), Greece (G), Italy (I), Lybia (L), Morocco (M), Pakistan (P), Spain (S), and Tunisia (T). **(D)** highlights the geographic origin of the Mediterranean accessions characterized by STRUCTURE analysis. **(E)** presents the three-dimensional scatter plot resulting from DAPC applied to k-means clusters.

The admixture model implemented by the software STRUCTURE (Pritchard et al., 2000) was used to infer population structure. In order to meet the model assumption of independence among loci, the SNP dataset was pruned for linkage disequilibrium, resulting in an input file of 1,196 markers. The application of the ΔK method (Evanno et al., 2005) revealed the presence of hierarchical structure, as models with two, three, and five genetic clusters (k) were all associated with high ΔK values (**Supplementary Figure S2**). At the finest level of structure (k = 5), the first cluster (named “SWM”) was predominant in the South-Western Mediterranean area and, in particular, in Algeria and Tunisia. The second cluster (named “SEM”) was predominant in the South-Eastern Mediterranean area, especially in Cyprus, Egypt, and Ethiopia. The third cluster (named “BM”) was frequent in both South-Western and North Mediterranean countries. The fourth cluster (named “NM”) occurred at high frequency in Northern Mediterranean countries. Finally, the fifth cluster (named “A”) was predominant in Asia. Nineteen accessions were classified as admixed, as they showed the highest cluster membership coefficient (q_i_) lower than 0.6 (**Figures 1A, D** and **Supplementary Figure S3**).

For k = 3, two groups were formed among accessions previously assigned to clusters SEM–BM and clusters NM–A, whereas for k = 2, a unique group was formed among accessions previously assigned to clusters SEM, BM, NM, and A (**Figures 1B, C** and **Supplementary Table S3**).

### Nonparametric Inference of Population Structure and Individual Genetic Relationships

The STRUCTURE algorithm is known to work suboptimally for hierarchical structure, and, in addition, it uses Hardy–Weinberg equilibrium for clustering, a condition that is hardly met in many crop species (Jombart et al., 2010; Lawson et al., 2018). Therefore, we complemented the investigation of genetic structure with the nonparametric method of k-means, which identifies clusters of individuals displaying the minimal within-group variance. Linkage disequilibrium-pruned SNPs were also used in this case, in order to avoid autocorrelation among linked loci. Based on the Bayesian Information Criterion, we found that genetic variation in the collection was efficiently summarized for a minimum number of clusters (k) equal to 5 (**Supplementary Figure S4**). Notably, clusters were identical to the ones identified by STRUCTURE for k = 5, except for the inclusion of accessions classified by STRUCTURE as admixed. Therefore, k-means clusters were named as previously mentioned (**Supplementary Table S3**).

Genetic relationships among k-means clusters were visually assessed by discriminant analysis of principal component analysis, which yields synthetic variables (discriminant functions) that maximize variation among groups while minimizing within-group variation (Jombart et al., 2010). The first three discriminant functions clearly differentiated the five k-means clusters, thus indicating the occurrence of population structure (**Figure 1E**). Hierarchical clustering highlighted a divergent lineage leading to cluster SWM and successive nodes separating clusters SEM–BM from clusters NM–A (Supplementary Fig. S5). Calculation of pairwise F_ST_ distances indicated high levels of differentiation among populations. The highest F_ST_ distance (0.34) was observed between cluster SWM and clusters SEM–BM, whereas the lowest distance (0.21) was observed between clusters NM and A (**Table 1**).

**Table 1:** F_ST_ distance matrix of k-means clusters identified in this study.

	Cluster SWM	Cluster SEM	Cluster BM	Cluster NM	Cluster A
Cluster SWM	0				
Cluster SEM	0.34	0			
Cluster BM	0.34	0.24	0		
Cluster NM	0.26	0.30	0.25	0	
Cluster A	0.31	0.28	0.27	0.21	0

Genetic relationships among individual accessions were finally assessed by the construction of a hierarchical tree based on the allele-sharing distance and the Ward’s clustering algorithm. In accordance with previous reports, genetic variation displayed by Mediterranean germplasm was higher than the one displayed by Asian accessions (**Supplementary Figure S6**).

### Correlation Between Population Structure and Phenotypic Traits

Average phenotypic traits (flowering time, plant height, height of the first flowering node, seed area, perimeter, and diameter) recorded for the nonredundant lentil accessions genotyped in this study are reported in the **Supplementary Table S3**, whereas mean, range, standard deviation, and broad-sense heritability associated with all the phenotypic traits are reported in the **Supplementary Table S4**. Significant genotypic effects on all the assessed phenotypic traits were detected by analysis of variance (**Supplementary Table S5**). Selection, especially concerning seed traits, is assumed to be one of the main factors driving the evolution of cereals and grain legumes (Preece et al., 2017). Therefore, we tested for association between k-means clusters and the morpho-agronomic traits assessed at the phenotypic level. Clusters SWM and BM were associated with significantly smaller and larger seed sizes, respectively, whereas no difference in seed size was found among clusters SEM, NM, and A (**Table 2** and **Supplementary Figure S7**). Clusters SWM and SEM were characterized by significantly anticipated flowering and lower height of the first flowering node compared with clusters BM, NM, and A (**Table 2**). Differences were also found for plant height, which was significantly higher and lower for clusters NM and SWM, respectively (**Table 2**). The distribution of seed phenotypic traits in individual clusters is shown in the **Supplementary Figure S8**.

**Table 2:** Plant and seed phenotypic traits (mean ± SD) assessed on the five lentil genetic clusters identified in this study.

Cluster	Flowering time (days)	Plant height (cm)	Height of first flowering node (cm)	Area (mm2)	Perimeter (mm)	Diameter (mm)
SWM	134^a^ ± 6.5	31^a^ ± 4.3	13^a^ ± 2.9	11.9^b^ ± 2.2	13.0^c^ ± 1.2	3.8^c^ ± 0.3
SEM	132^a^ ± 4.2	36^b^± 2.9	13^a^ ± 2.3	16.2^b,c^ ± 3.3	15.7^b^ ± 1.4	4.5^b^ ± 0.5
BM	143^b^ ± 3.8	41^b^± 4.7	18^b^ ± 3.1	25.6^a^± 4.3	19.1^a^ ± 1.6	5.6^a^ ± 0.5
NM	146^b^ ± 6.0	44^c^± 5.8	20^b^± 4.5	18.4^c^± 6.2	16.2^b^ ± 3.0	4.7^b^ ± 0.8
A	144 ^b^± 5.0	37^b^± 6.1	17^b^ ± 4.4	17.2^c^± 6.7	15.7^b^ ± 2.6	4.6^b^ ± 0.8

Different letters indicate significant differences (p < 0.05) Inferred by the Tukey’s honestly significant difference test.

### Detection of Distinctive Alleles

The pairwise fixation index (F_ST_) was computed at individual SNP loci in order to search for alleles that mostly differentiate each of the k-means clusters from the rest of the germplasm collection. The F_ST_ parameter reaches the upper limit of 1 when the two groups under comparison fix different alleles. As shown in **Supplementary Figure S9**, loci with F_ST_ > 0.8 could be assigned to all the clusters. In addition, alleles that are nearly private (F_ST_ > 0.95) for clusters SWM, SEM, and A were characterized. Overall, these results suggest the possibility to set up molecular keys enabling the assignment of genotypes to clusters and indicate polymorphisms that possibly arose from cluster-specific events of drift or selection.

### Marker–Phenotype Associations

A GWA analysis was carried out to look for associations with phenotypic traits, using a multi-locus regression model. After false discovery rate correction, significant associations were found for all the morphometric measurements related to seed size, namely, seed area, seed perimeter, and seed diameter. In addition, the same marker (TP6160) was significantly associated with all the three traits (**Supplementary Table S6**). No significant association was found for the other traits for which phenotypic data were available.

## Discussion

Here, we report the successful application of GBS for the study of genetic diversity in cultivated lentil. Sequencing of a 188-plex GBS library obtained with the *Ape*KI restriction enzyme yielded about 2.4 million reads per sample. This value matches those (2.4–2.5 million reads per sample) obtained by Wong et al. (2015), reporting the application of GBS to the study of genetic relationships among *Lens* species. However, these authors used a different GBS protocol for library preparation, based on the simultaneous use of two restriction enzymes, the rare cutter *Pst*I, and the frequent cutter *Msp*I. Following the SNP filtering procedure, more than 6.5 K polymorphisms were detected, suggesting that GBS could be conveniently used in lentil for further characterization of *ex situ* collections and genomic studies.

Population structure analysis of nonredundant accessions with parametric (STRUCTURE) and nonparametric (k-means) methods indicated that five genetic clusters (K = 5) could efficiently summarize patterns of variation in the data (**Supplementary Figures S2** and **S4**). Remarkably, the composition of clusters obtained by the two methods is identical, except for a few accessions classified as admixed by the STRUCTURE genetic model (**Supplementary Table S3**). The obtainment of high values of the ΔK *post hoc* statistics, estimating the plausibility of STRUCTURE clustering, not only for k = 5 but also for k = 2 and k = 3 (**Supplementary Figure S2**), can be explained by the occurrence of successive hierarchical levels of structure, which can be simultaneously detected by the STRUCTURE/ΔK methods (Evanno et al., 2005; Pavan et al., 2017a).

In accordance with other studies, our work highlights the occurrence of geographic stratification between the Mediterranean and Asian gene pools (**Supplementary Table S3**) (Ferguson et al., 1998; Lombardi et al., 2014; Khazaei et al., 2016). This is likely the outcome of distinct genetic drift events following the diffusion of lentil cultivation westward and eastward from its center of origin. In addition, peculiarities of the Mediterranean and Asian agro-climatic zones might have caused specific selection pressures leading to genetic differentiation (Khazaei et al., 2016). High level of genetic diversity was found within Mediterranean germplasm, consistent with previous reports (Erskine et al., 1989; Lombardi et al., 2014; Khazaei et al., 2016).

We identified strong genetic structure within the Mediterranean gene pool, as Mediterranean accessions were distributed across five genetic clusters (**Figure 1D**, **Supplementary Figure S3** and **Supplementary Table S3**). This is seemingly in contrast with the work of Khazaei et al. (2016), which groups Mediterranean germplasm in a single genetic cluster. However, Khazaei et al. (2016) aimed at investigating lentil genetic structure at a global scale, and it is known that the STRUCTURE/ΔK methods may not detect lower levels of hierarchical structure (Evanno et al., 2005; Lawson et al., 2018). In addition, the sampling strategy strongly influences the number of clusters inferred by the same methods (Lawson et al., 2018).

We could describe geographic patterns of variation in genetic structure (**Figure 1D** and **Supplementary Figure S3**). In particular, three clusters that occur at high frequency in South-Western Mediterranean (cluster SWM), South-Eastern Mediterranean (cluster SEM), and North Mediterranean (cluster NM) countries were found. Cluster SWM, mostly composed of Algerian and Tunisian accessions, might arise from the relatively recent (early XX century) introduction of lentil in these countries during the colonial period (Laumont, 1960; Gaad et al., 2018). Intense maritime trades among Ancient Greece, Cyprus, and Egypt have been amply documented (Bunson, 1999), so it is tempting to speculate that cluster SEM might be the result of the historical route that brought lentil cultivation to the Nile Valley. In accordance with this hypothesis, the diffusion of lentil from South-Eastern Europe to Egypt, and from Egypt to Ethiopia (through the Hamitic invaders), has been previously hypothesized (Robertson and Maxted, 2001). Our findings make less likely the hypothesis advanced by Fratini et al. (2011), stating that lentil cultivation reached Ethiopian highlands through the Arabian coast rather than by the Nile. Finally, cluster NM might group accessions most suitable to northern climates. This is consistent with the identification, by Khazaei et al. (2016), of a lentil genetic cluster mostly present in temperate countries.

Different from clusters SWM, SEM, and NM, cluster BM displayed a wide geographic distribution across Mediterranean regions (**Figure 1D**). This result might be due to the extensive exchange of germplasm with peculiar phenotypic features (larger seed size in particular, as shown in **Table 2** and **Supplementary Figure S8**).

**Figure 1D** and **Supplementary Figure S3** show that, although there is clear difference in the relative frequency of genetic clusters in different zones of the Mediterranean basin, different clusters may coexist in the same geographical area. This can be explained by the cleistogamous nature of lentil (Wilson, 1972), which limits to sporadic outbreeding events gene flow among clusters introduced in the same area. According to this scenario, admixed accessions should mostly derive from artificial crosses. However, it has been stressed that admixture relationships identified by the STRUCTURE algorithm do not necessarily reflect interbreed between ancestral populations (Lawson et al., 2018).

Remarkably, our study clearly shows correlation between genetic structure and phenotypic diversity in lentil (**Table 2**), indicating that selection played a major role in determining genetic divergence among populations. Population structure was strongly correlated with seed size (**Table 2**), similar to what has been shown in faba bean (*Vicia faba* L.) and chickpea (*Cicer arietinum* L.) (Göl et al., 2017; De Giovanni et al., 2017; Pavan et al., 2017b), thus suggesting that similar anthropic selection pressures shaped the evolution of different grain legume species. We highlight that the classification proposed from Barulina et al. (1930), dividing cultivated lentil according to the seed diameter in *microsperma* (<6 *mm*) and *macrosperma* (>6 mm), would not efficiently distinguish the genetic clusters identified in this study, which were associated with average seed diameters ranging from 3.8 mm (cluster SWM) to 5.6 mm (cluster BM) (**Table 2**). Correlation with genetic structure was also found for earliness. In particular, early flowering and lower height of the first flowering node were displayed by two clusters (SWM and SEM) occurring with high frequency in the Southern Mediterranean Basin (**Table 2**, **Figure 1D** and **Supplementary Figure S3**). This suggests the selection for an adaptive trait that increases the probability to escape extreme heat and aridity occurring in the end of the crop cycle.

Phenotypic characterization of the genetic clusters identified in this study might help to further define cluster-specific traits, related for example to seed chemical composition and tolerance/resistance to stresses. This kind of information is of main interest to choose appropriate parental lines for hybridization. In addition, crossing parental genotypes from genetically distant clusters in breeding programs increases the probability of selecting favorable recombinants (Roy et al., 2013), although outbreeding depression, due to the disruption of allelic combinations providing adaptation to specific agro-ecosystems, cannot be excluded (Schierup and Christiansen, 1996). Given these premises, it will be important to assign uncharacterized lentil germplasm to a specific genetic cluster. With this respect, knowledge on the germplasm geographical origin or phenotype might not be sufficient, as: 1) despite the occurrence of geographical patterns of variation, different clusters might coexist in the same geographical area (**Figure 1D** and **Supplementary Figure S3**); different clusters might have overlapping phenotypes (**Table 2**, **Supplementary Table S3** and **Supplementary Figure S8**). Importantly, the calculation of F_ST_ distances at individual SNP loci highlighted several alleles that are nearly cluster specific (**Supplementary Figure S9**), thus indicating the possibility to set up molecular keys to assign lentil germplasm to a specific cluster. Besides this, some of the loci displaying high F_ST_ values might be the result of specific events of selection leading to phenotypic differentiation of lentil populations.

A preliminary GWA study was performed merging genotypic and phenotypic data. Five significant associations were found, and, in addition, the same marker was found to be associated with all the traits related to seed size (**Supplementary Table S6**), indicating correlation among traits. The upcoming public release of the lentil genome sequence will allow to relate markers with specific genomic regions and verify whether these regions overlap with quantitative trait loci previously characterized for seed size (Fedoruk et al., 2013; Verma et al., 2015; Khazaei et al., 2018).

## Conclusion

GBS proved to be a powerful tool for the cost-effective identification of DNA polymorphisms in lentil. We identified genetic clusters of great interest for conservation genetics and breeding and found relationships between population structure, geographical origin, and phenotypic traits. Finally, we found molecular keys to assign germplasm to specific clusters. Information provided by this study might be integrated by further characterization of lentil *ex situ* collections, provided that raw sequencing data are publicly available. We are currently performing an extensive agronomic, nutritional, and technological characterization of the lentil collection genotyped in this work, aiming to identify and map valuable alleles through GWA studies.

## Data Availability

The datasets generated for this study can be found in NCBI-SRA, SRR8863759.

## Author Contributions

SP, CL, CM, CDG, and LR conceived the research. NB, SP, VF, AM, and GM carried out lab and field experiments. SP, FT, and DC analyzed SNP data. SP wrote the manuscript.

## Funding

This research has been performed within the project “LEgume GEnetic REsources as a tool for the development of innovative and sustainable food TEchnological system” supported under the “Thought for Food” Initiative by Agropolis Fondation (through the “*Investissements d’avenir*” programme with reference number ANR-10-LABX-0001-01”), Fondazione Cariplo, and Daniel & Nina Carasso Foundation.

## Conflict of Interest Statement

The authors declare that the research was conducted in the absence of any commercial or financial relationships that could be construed as a potential conflict of interest.
