# Supplementary material for: Genotyping by Sequencing of Cultivated Lentil (Lens culinaris Medik.) Highlights Population Structure in the Mediterranean Gene Pool Associated With Geographic Patterns and Phenotypic Variables
Source: Front Genet. 2019 Sep 18;10:872. doi: 10.3389/fgene.2019.00872 (PMC6759463; doi:10.3389/fgene.2019.00872)
Supplement: Supplementary file 1 [file Presentation_1.pptx]

## Slide 1
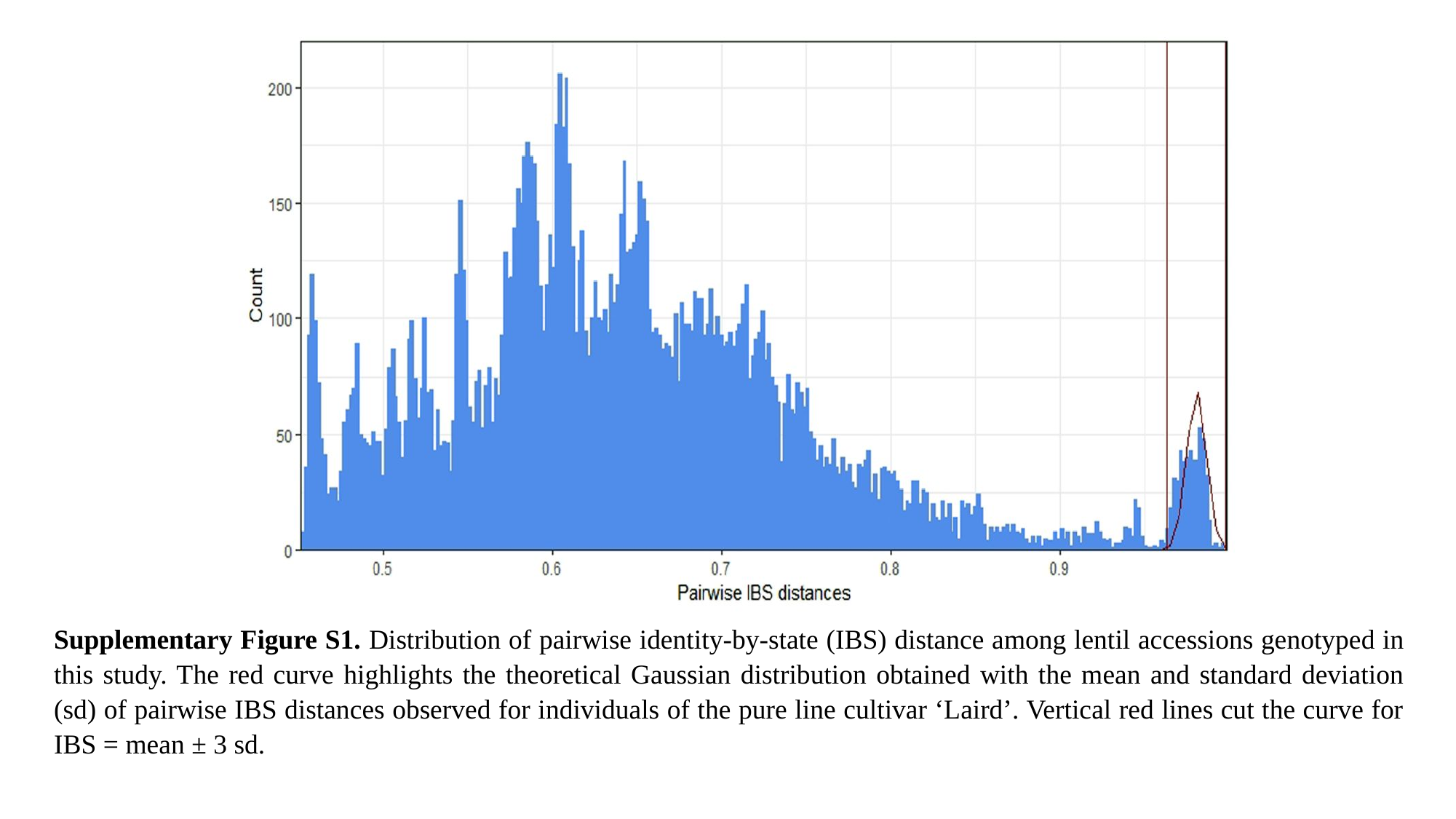

Supplementary Figure S1. Distribution of pairwise identity-by-state (IBS) distance among lentil accessions genotyped in this study. The red curve highlights the theoretical Gaussian distribution obtained with the mean and standard deviation (sd) of pairwise IBS distances observed for individuals of the pure line cultivar ‘Laird’. Vertical red lines cut the curve for IBS = mean ± 3 sd.
