# Supplementary material for: Genotyping by Sequencing of Cultivated Lentil (Lens culinaris Medik.) Highlights Population Structure in the Mediterranean Gene Pool Associated With Geographic Patterns and Phenotypic Variables
Source: Front Genet. 2019 Sep 18;10:872. doi: 10.3389/fgene.2019.00872 (PMC6759463; doi:10.3389/fgene.2019.00872)
Supplement: Supplementary file 3 [file Presentation_3.pptx]

## Slide 1
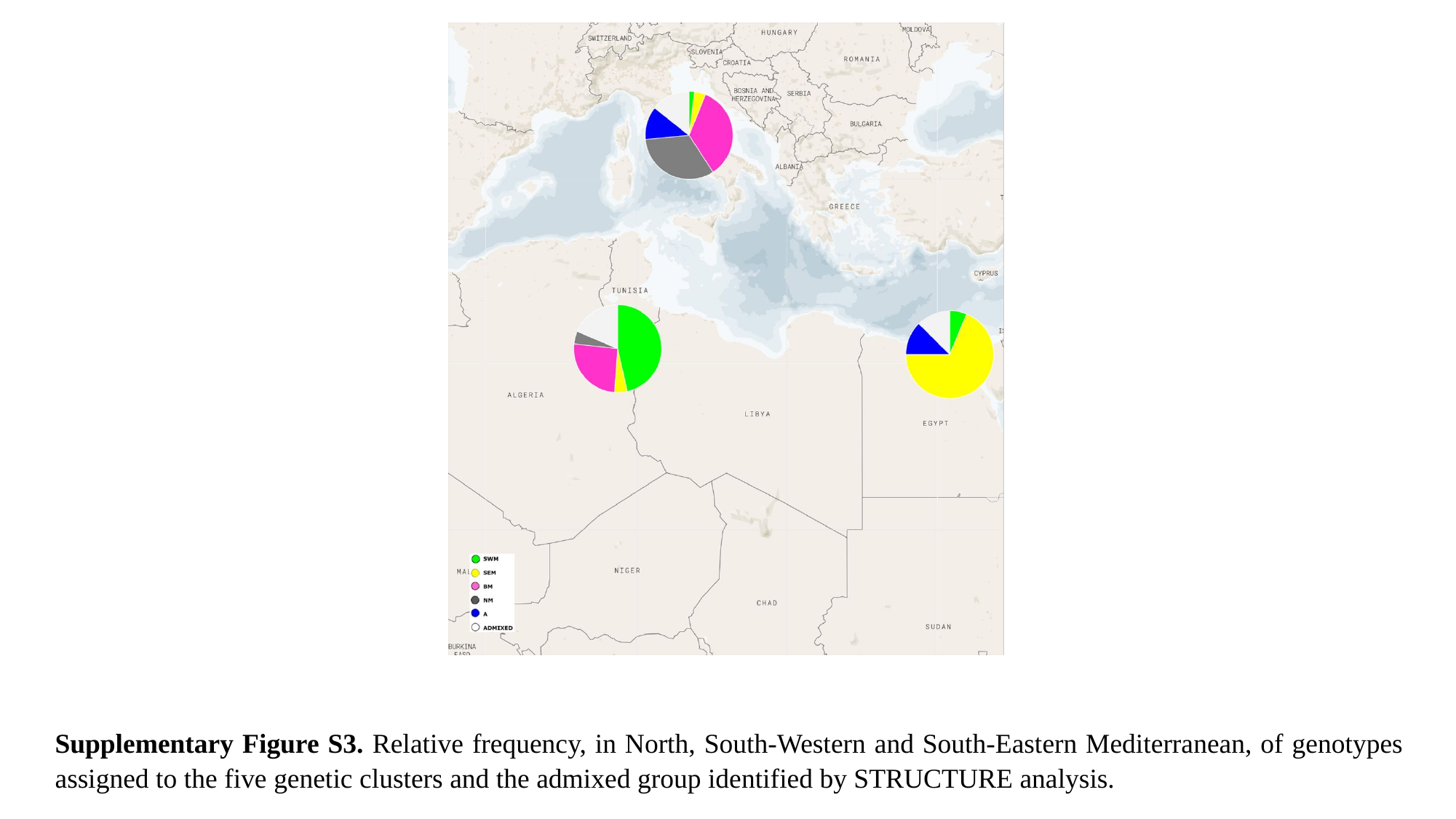

Supplementary Figure S3. Relative frequency, in North, South-Western and South-Eastern Mediterranean, of genotypes assigned to the five genetic clusters and the admixed group identified by STRUCTURE analysis.
