# Supplementary figures and images for: Genotyping by Sequencing of Cultivated Lentil (Lens culinaris Medik.) Highlights Population Structure in the Mediterranean Gene Pool Associated With Geographic Patterns and Phenotypic Variables
Source: Front Genet. 2019 Sep 18;10:872. doi: 10.3389/fgene.2019.00872 (PMC6759463; doi:10.3389/fgene.2019.00872)

## Slide 1
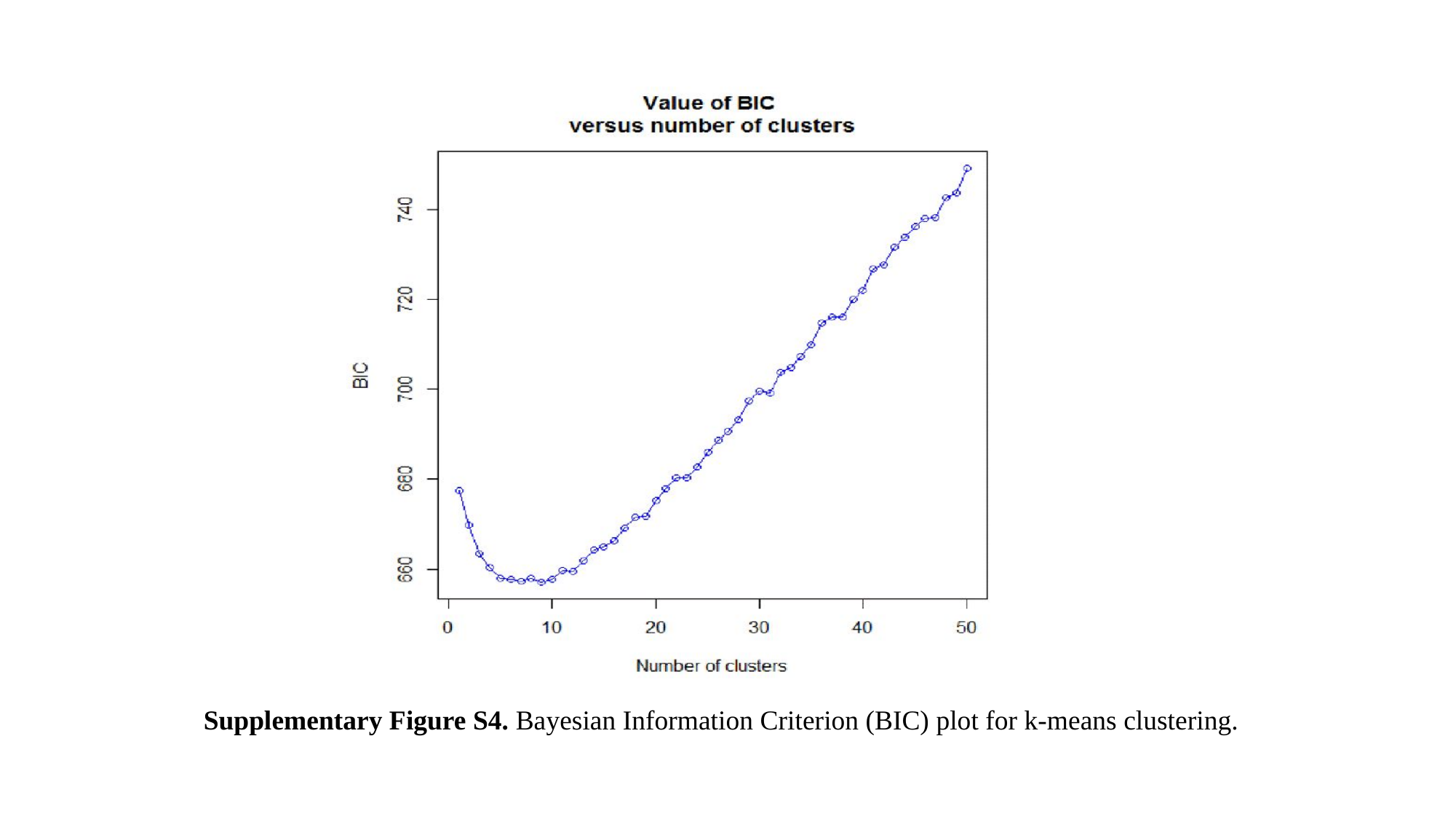

Supplementary Figure S4. Bayesian Information Criterion (BIC) plot for k-means clustering.

Supplement: Supplementary file 4 [file Presentation_4.pptx]
