# Supplementary material for: Genotyping by Sequencing of Cultivated Lentil (Lens culinaris Medik.) Highlights Population Structure in the Mediterranean Gene Pool Associated With Geographic Patterns and Phenotypic Variables
Source: Front Genet. 2019 Sep 18;10:872. doi: 10.3389/fgene.2019.00872 (PMC6759463; doi:10.3389/fgene.2019.00872)
Supplement: Supplementary file 5 [file Presentation_5.pptx]

## Slide 1
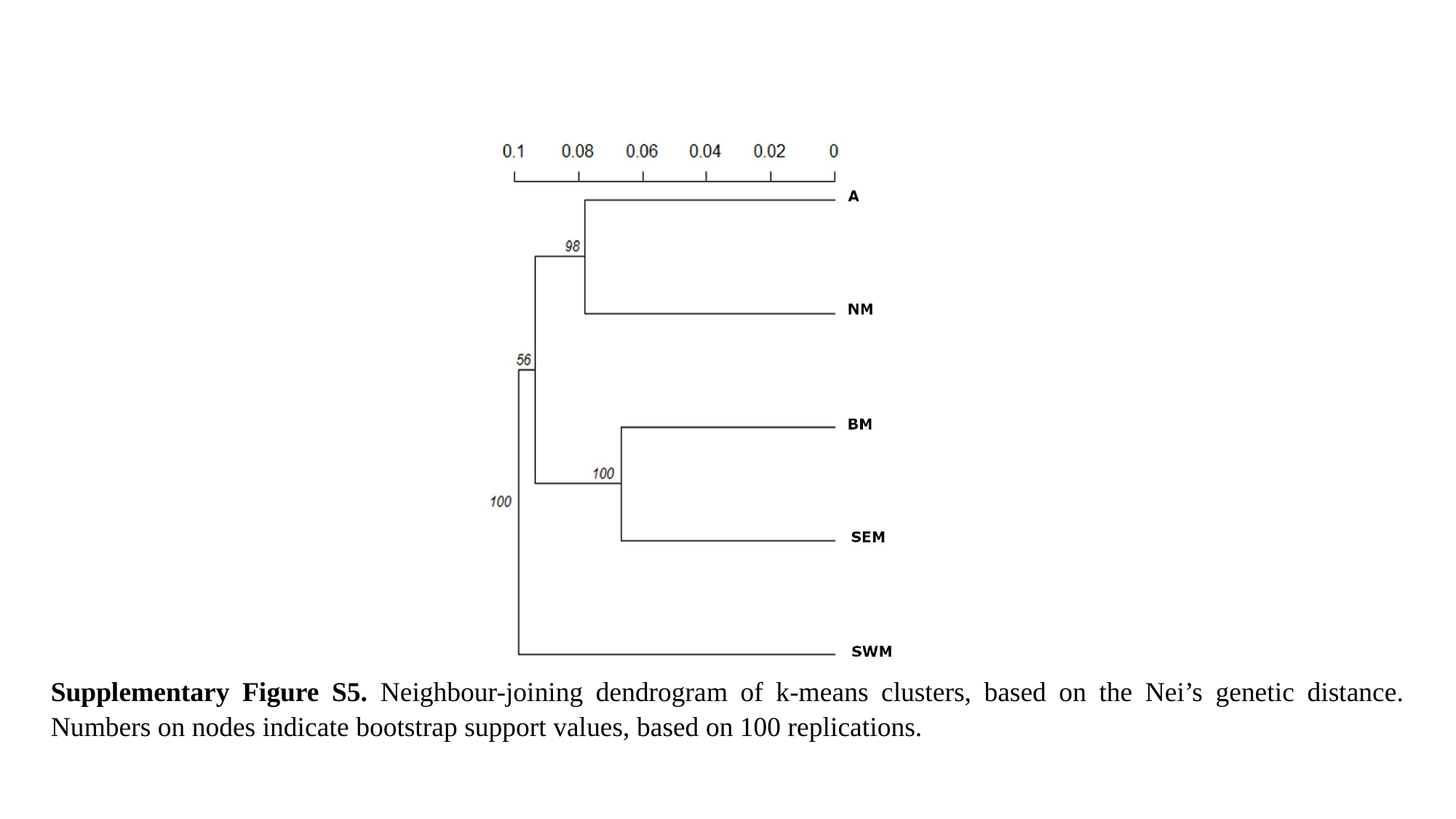

Supplementary Figure S5. Neighbour-joining dendrogram of k-means clusters, based on the Nei’s genetic distance. Numbers on nodes indicate bootstrap support values, based on 100 replications.
