# Supplementary material for: Genotyping by Sequencing of Cultivated Lentil (Lens culinaris Medik.) Highlights Population Structure in the Mediterranean Gene Pool Associated With Geographic Patterns and Phenotypic Variables
Source: Front Genet. 2019 Sep 18;10:872. doi: 10.3389/fgene.2019.00872 (PMC6759463; doi:10.3389/fgene.2019.00872)
Supplement: Supplementary file 7 [file Presentation_7.pptx]

## Slide 1
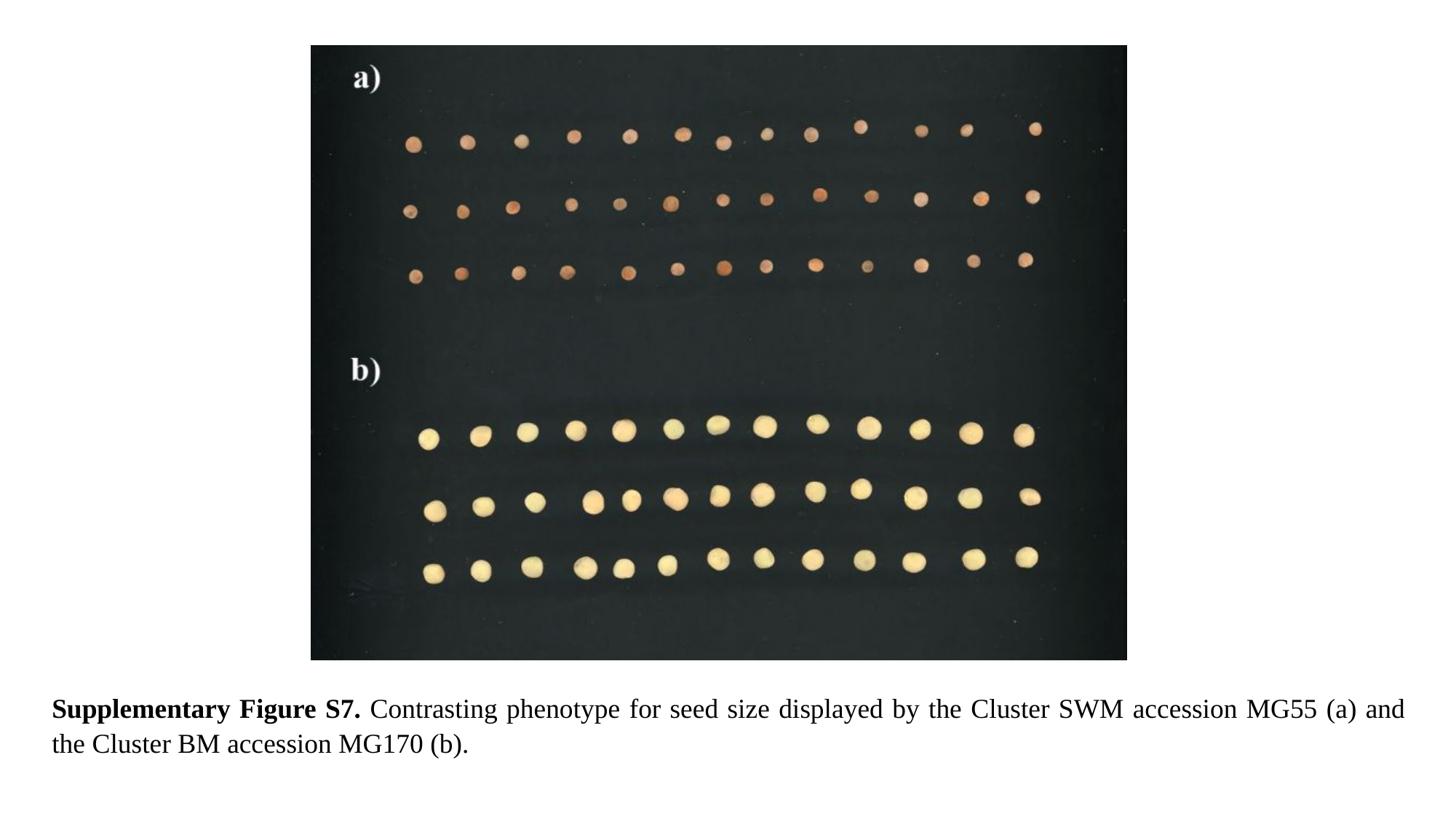

Supplementary Figure S7. Contrasting phenotype for seed size displayed by the Cluster SWM accession MG55 (a) and the Cluster BM accession MG170 (b).
