# Supplementary material for: Genotyping by Sequencing of Cultivated Lentil (Lens culinaris Medik.) Highlights Population Structure in the Mediterranean Gene Pool Associated With Geographic Patterns and Phenotypic Variables
Source: Front Genet. 2019 Sep 18;10:872. doi: 10.3389/fgene.2019.00872 (PMC6759463; doi:10.3389/fgene.2019.00872)
Supplement: Supplementary file 8 [file Presentation_8.pptx]

## Slide 1
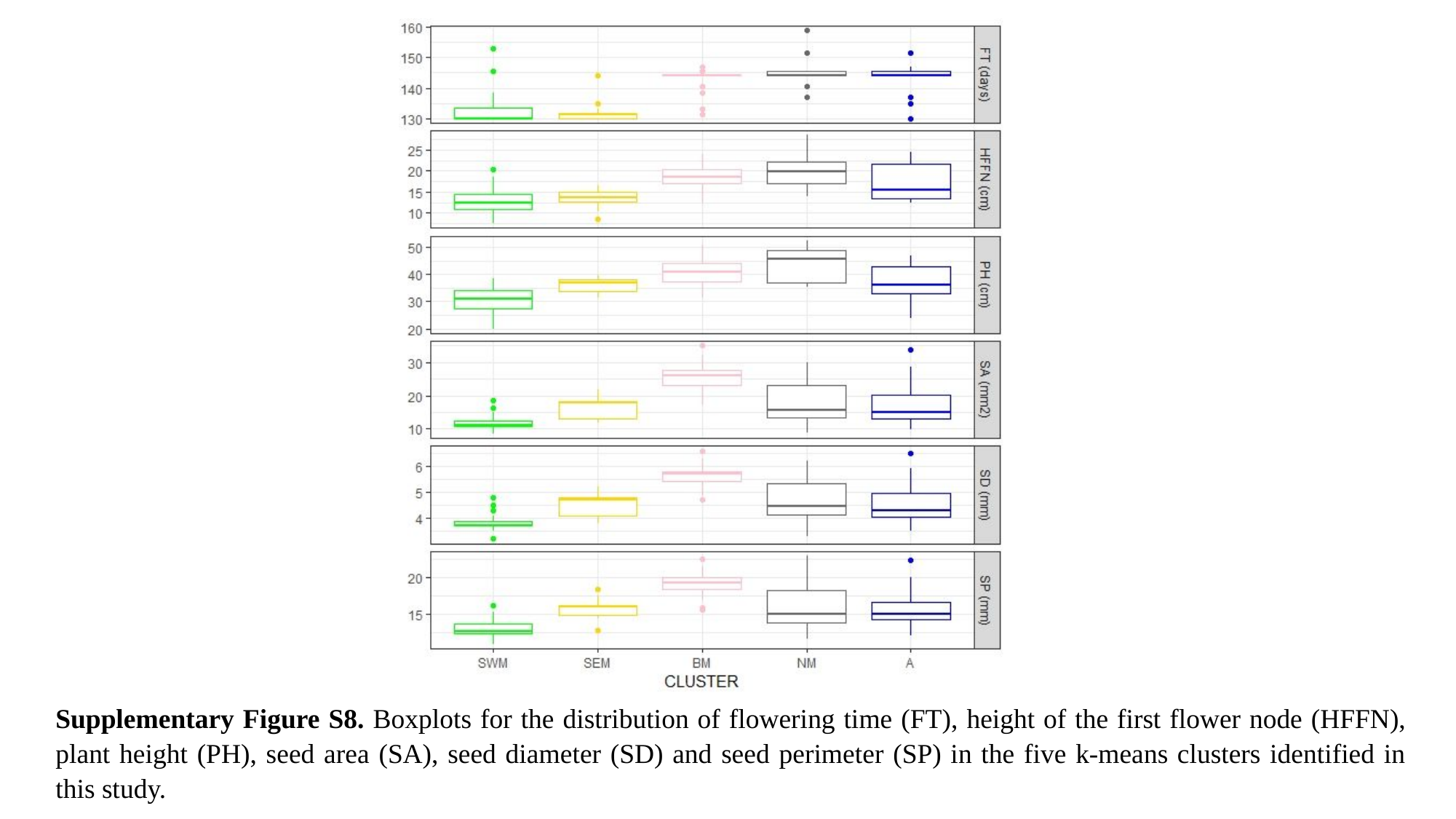

Supplementary Figure S8. Boxplots for the distribution of flowering time (FT), height of the first flower node (HFFN), plant height (PH), seed area (SA), seed diameter (SD) and seed perimeter (SP) in the five k-means clusters identified in this study.
