# Supplementary material for: Genotyping by Sequencing of Cultivated Lentil (Lens culinaris Medik.) Highlights Population Structure in the Mediterranean Gene Pool Associated With Geographic Patterns and Phenotypic Variables
Source: Front Genet. 2019 Sep 18;10:872. doi: 10.3389/fgene.2019.00872 (PMC6759463; doi:10.3389/fgene.2019.00872)
Supplement: Supplementary file 9 [file Presentation_9.pptx]

## Slide 1
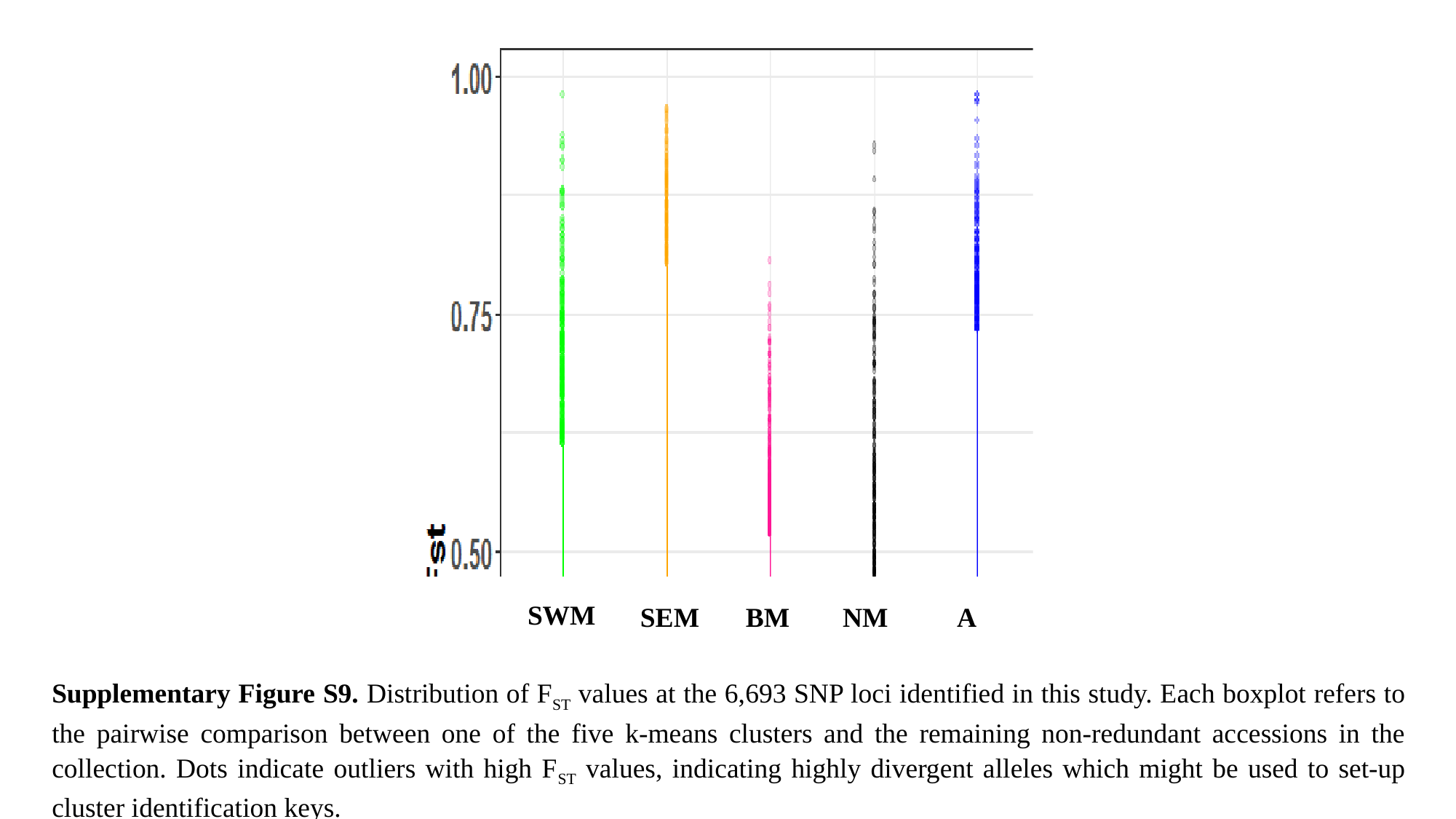

SWM
SEM
BM
NM
A
Supplementary Figure S9. Distribution of FST values at the 6,693 SNP loci identified in this study. Each boxplot refers to the pairwise comparison between one of the five k-means clusters and the remaining non-redundant accessions in the collection. Dots indicate outliers with high FST values, indicating highly divergent alleles which might be used to set-up cluster identification keys.
